# Hemostasis using a novel self-assembling peptide for a bleeding gastric hyperplastic polyp after endoscopic resection

**DOI:** 10.1055/a-2418-0891

**Published:** 2024-10-08

**Authors:** Yo Kubota, Risako Sudo, Kenji Ishido, Chika Kusano

**Affiliations:** 1 38088 Gastroenterology, Kitasato University School of Medicine, Sagamihara, Japan

Endoscopic resection (ER) is considered when a gastric hyperplastic polyp (HPP) leads to bleeding or anemia 1 . However, achieving hemostasis for bleeding from a gastric HPP after ER can be challenging, depending on the lesion location and the patient’s clinical background. PuraStat (3-D Matrix Ltd., Tokyo, Japan), a novel self-assembling peptide, has been utilized as a hemostatic agent in various endoscopic procedures 2 3 4 5 . We report a case of successful hemostasis using PuraStat for post-ER bleeding from a gastric HPP.

A 79-year-old woman was hospitalized to receive steroid therapy (50 mg prednisolone/day) for polymyositis. However, she developed tarry stools, and blood tests revealed anemia with a hemoglobin level of 8.4 g/dL. She had an active *Helicobacter pylori* infection but was not on antithrombotic therapy. An emergency endoscopy identified a gastric HPP with persistent bleeding from the greater curvature of the cardia ( Fig. 1 ). ER was performed, but persistent bleeding was observed from the post-ER ulcer. Efforts to achieve hemostasis using forceps and local epinephrine injections were unsuccessful due to significant movement during breathing and the ulcer location in the greater curvature of the cardia. Clipping with the SureClip (Micro-Tech Co., Ltd., Nanjing, China) was attempted, but bleeding persisted from the gap between two SureClips. Additionally, the mucosa at the ulcer's base was extremely fragile, leaving no space to place another SureClip. Consequently, PuraStat was applied to achieve hemostasis ( Video 1 ). The bleeding slowed immediately upon PuraStat application, and complete hemostasis was achieved within 1 min. A second-look endoscopy revealed residual PuraStat at the ulcer base, confirming sustained hemostasis ( Fig. 2 ). The patient resumed oral intake and was subsequently discharged.

**Fig. 1:**
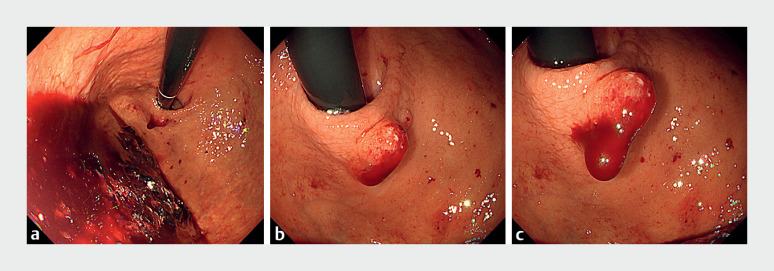
**a–c** Emergency endoscopy revealing a large number of coagula with fresh blood in the stomach ( **a** ), a gastric hyperplastic polyp (HPP) located in the greater curvature of the cardia ( **b** ), and persistent bleeding from the gastric HPP ( **c** ).

**Fig. 2:**
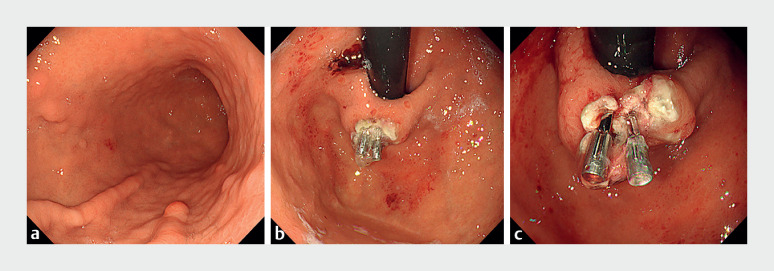
A second-look endoscopy was performed. **a** Second-look endoscopy showing that the gastric coagula had disappeared. **b** Post-endoscopic resection ulceration was observed in the hyperplastic polyp located in the greater curvature of the cardia, and hemostasis was confirmed. **c** PuraStat, along with SureClip, was observed at the bottom of the ulcer.

Hemostasis was achieved using a novel self-assembling peptide (PuraStat) for bleeding from a gastric hyperplastic polyp after endoscopic resection.Video 1

Therefore, we found that the extensive use of PuraStat is effective in managing persistent bleeding from gastric HPP after ER, particularly when accessing the bleeding site and achieving hemostasis are challenging.

Endoscopy_UCTN_Code_TTT_1AO_2AD

## Notes

Endoscopy E-Videos https://eref.thieme.de/e-videos
*E-Videos* is an open access online section of the journal *Endoscopy* , reporting on interesting cases and new techniques in gastroenterological endoscopy. All papers include a high-quality video and are published with a Creative Commons CC-BY license. Endoscopy E-Videos qualify for HINARI discounts and waivers and eligibility is automatically checked during the submission process. We grant 100% waivers to articles whose corresponding authors are based in Group A countries and 50% waivers to those who are based in Group B countries as classified by Research4Life (see: https://www.research4life.org/access/eligibility/ ). This section has its own submission website at https://mc.manuscriptcentral.com/e-videos .

## References

[1] Kato M Ota H Okuda M Guidelines for the management of Helicobacter pylori infection in Japan: 2016 revised edition Helicobacter 2019 24 e12597 10.1111/hel.12597 31111585

[2] Uraoka T Ochiai Y Fujimoto A A novel fully synthetic and self-assembled peptide solution for endoscopic submucosal dissection-induced ulcer in the stomach Gastrointest Endosc 2016 83 1259 1264 10.1016/j.gie.2015.11.015 26608126

[3] de Nucci G Reati R Arena I Efficacy of a novel self-assembling peptide hemostatic gel as rescue therapy for refractory acute gastrointestinal bleeding Endoscopy 2020 52 773 779 10.1055/a-1145-3412 32316041

[4] Subramaniam S Kandiah K Chedgy F A novel self-assembling peptide for hemostasis during endoscopic submucosal dissection: a randomized controlled trial Endoscopy 2021 53 27 35 32679602 10.1055/a-1198-0558

[5] Yamaguchi D Ishida S Nomura T Endoscopic hemostasis of spurting colonic diverticular bleeding using the combination of self-assembling peptide solution and endoscopic band ligation Endoscopy 2023 55 E418 E419 10.1055/a-2008-0599 36758630 PMC9911289

